# Caregiver burden among informal caregivers of stroke survivors in Harare, Zimbabwe

**DOI:** 10.4102/sajp.v80i1.2080

**Published:** 2024-11-26

**Authors:** Farayi Kaseke, Lovemore Gwanzura, Timothy Kaseke, Cuthbert Musarurwa, Elizabeth Gori, Tawanda Nyengerai, Aimee Stewart

**Affiliations:** 1 Department of Physiotherapy, College of Medicine and Health Sciences, University of Rwanda, Kigali, Rwanda; 2 Department of Rehabilitation, Faculty of Medicine and Health Sciences, University of Zimbabwe, Harare, Zimbabwe; 3 Department of Laboratory Diagnostic and Investigative Sciences, Faculty of Medicine and Health Sciences, University of Zimbabwe, Harare, Zimbabwe; 4 Biomedical Research and Training Institute, Harare, Zimbabwe; 5 Department of Christian Counselling, World Bible School University, Joplin, Missouri, United States of America; 6 Department of Commerce, University of Zimbabwe, Harare, Zimbabwe; 7 Department of Biomedical Laboratory Sciences, College of Medicine and Health Sciences, University of Rwanda, Kigali, Rwanda; 8 Department of Medical Biochemistry, Molecular Biology and Genetics, College of Medicine and Health Sciences, University of Rwanda, Kigali, Rwanda; 9 Department of Veterinary Biosciences, Faculty of Veterinary Science, University of Zimbabwe, Harare, Zimbabwe; 10 The Best Health Solutions, Johannesburg, South Africa; 11 Department of Physiotherapy, University of the Witwatersrand, Johannesburg, South Africa

**Keywords:** stroke, caregiver burden strain, longitudinal study, stroke survivor outcomes, anxiety, depression, Zimbabwe

## Abstract

**Background:**

Stroke presents significant challenges for both survivors and caregivers, particularly in resource-limited settings like Zimbabwe. Identifying factors contributing to caregiver burden strain (CBS) is crucial to enhance support strategies.

**Objectives:**

This longitudinal study identified caregiver and stroke survivor characteristics associated with CBS among caregivers in Harare, Zimbabwe.

**Method:**

Altogether 188 stroke survivors and their caregivers participated with CBS assessed at 3 months and 12 months using the Caregivers Strain Index. Multiple linear regression was used to evaluate the association of explanatory variables with CBS. Model fit was evaluated using the Akaike’s Information Criterion and *R*^2^.

**Results:**

Caregivers experiencing anxiety or depression showed increased CBS at 3 months (β = 2.46, *p* < 0.001) and 12 months (β = 2.73, *p* = 0.016). Work adjustments were associated with higher CBS at 3 months (β = 3.84, *p* < 0.001). Caregivers feeling overwhelmed had significantly higher CBS at 3 months (β = 3.36, *p* < 0.001). Stroke survivors’ poor physical outcomes and reliance on health insurance were associated with CBS at 12 months (β = 4.34, *p* = 0.006). Caring for married stroke survivors was associated with reduced CBS (β = –2.83, *p* < 0.001).

**Conclusion:**

Caregiver anxiety, depression, work adjustments and poor physical and social outcomes in stroke survivors contributed to increased CBS. Targeted interventions addressing mental health and social support are essential to reduce CBS.

**Clinical implications:**

Multifaceted interventions that address caregiver mental health and social support are vital to reduce CBS and improve outcomes in resource-constrained settings like Zimbabwe.

## Introduction

Stroke is a leading cause of disability worldwide, placing a considerable burden on both stroke survivors and their caregivers (Bakas et al. 2014). After a stroke, 60% – 80% of survivors require caregivers (formal (paid) or informal (unpaid)) because of physical and cognitive limitations (Bakas et al. 2014; Çakir et al. 2015; Lutz & Young 2010a). Informal caregivers typically include family members, predominantly women, and also include children, spouses or other relatives, who form the primary support system post hospital discharge (Grant, Hunt & Steadman 2014; Penido et al. 2017; Roopchand-Martin & Creary-Yan 2014). The present study enrolled informal caregivers as participants. These caregivers play a crucial role in providing physical, emotional and social support to stroke survivors during their recovery process (Gallagher-Thompson & Coon 2020; King, Hartke & Houle 2010; National Alliance for Caregiving 2015). Caregiving can be demanding and stressful, often leading to caregiver burden strain (CBS), which encompasses feelings of stress, strain and emotional distress resulting from the caregiving role (Patel et al. 2013; Pinquart & Sörensen 2003). Caregiver burden strain has been associated with adverse health outcomes for both caregivers and stroke survivors, including increased risk of depression, anxiety and poorer quality of life (Carod-Artal, Egido & González 2000; Haley et al. 2011). Given resource limitations, support for caregivers enabling home rehabilitation becomes essential in low-resourced countries. Female caregivers often face greater caregiving demands than males as they spend more time on concurrent caregiving tasks alongside other family and household responsibilities (Hsiao 2010; Lin, Fee & Wu 2012; Penido et al. 2017; Rhoda et al. 2015; Simon, Kumar & Kendrick 2009). Moreover, community support for caregivers of stroke survivors can reduce stroke recurrence, improve function, physical health and community reintegration, potentially reducing disability and depression among stroke survivors (Kalra et al. 2004; Farahani et al. 2020; Kamalakannan et al. 2016; Gallagher-Thompson & Coon 2019; Rahman & Salek 2016). Addressing caregivers’ perceived needs is crucial to improving their quality of life and reducing the burden of long-term caregiving.

Understanding the factors contributing to CBS following stroke is essential for developing effective support and intervention strategies aimed at mitigating CBS and improving the well-being of both caregivers and stroke survivors (Janssen et al. 2014). The impact of stroke extends beyond the individual stroke survivor to their family members and caregivers who often experience significant physical, emotional and financial strain as a result of their caregiving responsibilities (Garnett et al. 2022; Patel et al. 2023). Previous research has identified various factors associated with caregiver burden (CB) following stroke, including caregiver demographics, stroke survivor characteristics and the caregiving context (Pinquart & Sörensen 2003). For example, caregivers who are female, younger, unemployed and providing care for longer durations have been reported to experience higher levels of burden (Carod-Artal et al. 2000; Lin et al. 2012; Rhoda et al. 2015). Similarly, patient factors such as severity of stroke, functional impairment and cognitive deficits have been linked to increased CB (Haley et al. 2011). Additionally, social support networks, coping strategies and access to resources play a crucial role in influencing caregiver burden outcomes (King et al. 2010).

While literature provides valuable insights into the determinants of caregiver burden following stroke, there is a need for further research to explore these factors within the context of low- and middle-income countries (LMICs), where the burden of stroke is disproportionately high and resources for caregiving support are often limited (Bakas et al. 2014). Additionally, longitudinal studies examining changes in caregiver burden over time are essential for understanding the dynamic nature of caregiver burden and identifying critical time points for intervention (Patel et al. 2013). Stroke severity is directly associated with CBS, with stroke survivors’ negative perceptions about the future contributing to caregiver burden.

Our study aimed to investigate caregiver and stroke survivor factors associated with CBS following stroke in Harare, Zimbabwe, a low-income country with a high burden of stroke and limited resources for caregiving support. Specifically, we assessed caregiver burden at baseline and at 3-month and 12-month intervals post-stroke and explored the impact of caregiver demographics, stroke survivor characteristics and social support networks on caregiver burden outcomes. By elucidating the determinants of caregiver burden in this context, our study aims to inform the development of targeted interventions aimed at supporting caregivers and improving outcomes of survivors of stroke survivors in LMICs.

The data analysed and described in this study were collected as part of a PhD thesis (Kaseke 2021). Understanding the longitudinal impact of caregiving on CBS is crucial for developing effective interventions. Previous publications from the PhD thesis provided foundational knowledge on caregiver needs and caregiver training, while the current study offers a comprehensive analysis of how these needs translate into CBS over time. The findings from this prospective study can inform the refinement of caregiver support programs and policies, making a significant contribution to the literature on long-term CBS management. This study fills a gap in the existing research by providing empirical evidence on the progression of CBS, which was not covered in the initial needs assessment study.

## Research methods and design

### Study settings

**FIGURE 1:**
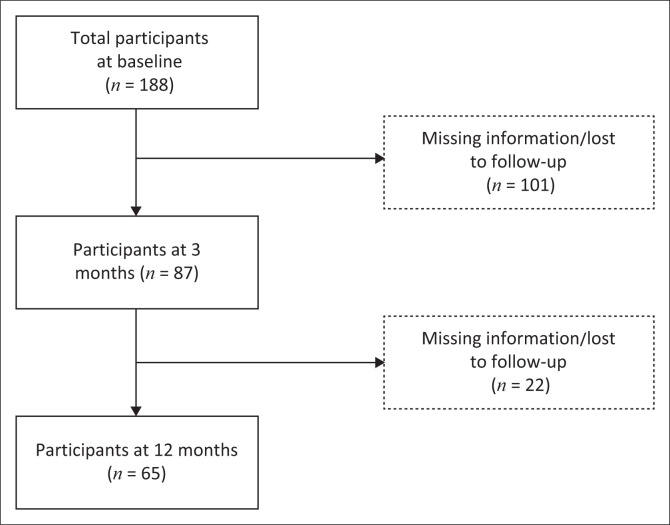
Flow of participants through the study.

In this study, we aimed to determine the factors associated with CBS among caregivers of stroke survivors in Harare, Zimbabwe. Sample size depicted a 2.5 change in EuroQol-5 dimension (EQ-5D) and a 1-point change in the Caregiver Strain Index (CSI) and a level of significance of 0.05 (Kalra et al. 2004). A total of 188 stroke survivor–caregiver dyads were consecutively recruited at baseline from three public referral hospitals in Harare, Zimbabwe: Parirenyatwa Group of Hospitals, Chitungwiza Central Hospital and Sally Mugabe Central Hospital based on admission to reduce selection bias. The analysis at 3 months included data from 87 caregivers and their respective wards who had survived stroke. Data for 101 caregiver-survivor dyads were excluded because of missing information on the desired outcome variable (CBS). Data were collected from caregivers only if the stroke survivor was alive and both could be found on follow-up. Similarly, at 12 months, data from only 65 caregivers and their stroke survivors were analysed (Figure 1). The inclusion criteria for caregivers were 18 years of age or older, designation as primary caregivers during the stroke survivor’s recuperation period and willingness to participate in the study. Stroke survivors were eligible for enrolment if they were diagnosed with stroke within the last 3 days and had a confirmed diagnosis by a healthcare professional.

### Study design

A longitudinal prospective study was carried out. Consecutive sampling was used to recruit participants from the aforementioned hospitals in Harare. Eighty four (44.6%) stroke survivors died during the 1 year period. Seventy one died during the first 3 months post-stroke and 13 after 3 months post-stroke. Once the survivor died, the caregiver was excluded from the follow-up. Loss to follow-up was 20%. To minimise loss to follow-up, we recorded the caregivers’ domicile addresses and at least three contact details. We made several calls 2 weeks before date of follow-up and carried out physical visits. However, some of the survivors and their caregivers had moved to the rural areas because of changes in employment status or they had gone back to their original place of residence although during the recruitment phase, they had indicated the given addresses as the permanent ones. We then realised that they had used relatives’ addresses to get admitted into the study settings and once better, they went back rendering it difficult to trace them; therefore, we excluded their data from analysis (Figure 1).

### Data collection

Baseline data were collected within 1 week of the stroke diagnosis when the stroke survivors were still admitted into hospital, and follow-up assessments were conducted at 3- and 12-months post-stroke. Trained research assistants conducted face-to-face interviews with caregivers to collect sociodemographic information related to both the caregiver and the stroke survivor. The CSI designed in either English or Shona (a local vernacular language) depending on the caregiver’s preference was administered by the research assistants to measure CBS. Caregivers were identified during hospital visiting times within 3 days of stroke survivor admission. The stroke survivor visitors were asked for at least two primary informal caregivers post-discharge for the patient if they were available; this was because, in the study setting, people normally tend to an ill relative in turns. In case, only one would be caregiving, then they would be recruited. Written informed consent was obtained from the prospective caregivers. To cater for any attrition, physical addresses and at least three contact details were recorded from each caregiver participants. In the case where there were two caregivers, information about both were recorded. Upon follow-up at 3 months and at 12 months, the caregiver who was tending to the survivor was interviewed. Caregiver participants were interviewed at follow-up only if the stroke survivor was still alive. All the participants were recorded and prospective follow-up dates communicated. The caregiver participant was phoned 2 weeks before the date of follow-up to book an appointment. Follow-up interviews were conducted ±2 weeks of appointment dates. The CSI consists of a series of 13 questions from which a score is derived. The original scale had good internal consistency (Cronbach’s alpha = 0.86) (Robinson 1983), similar to those found in a Turkish sample (α = 0.73 and α = 0.77), in two different moments by Ugur and Fadiloglu (2010).

### Study variables

#### Outcome variable

The outcome variable was CBS, assessed using the CSI. Total CSI scores were measured at 3 months and 12 months, aggregating scores from the 13 CSI questions. The expected overall score was 13. The total score for each caregiver was used to quantify the level of strain experienced. A total score > 7 was indicative of a high level of strain (Robinson 1983).

#### Caregivers’ demographic variables

The demographic data collected from caregivers encompassed a range of variables aimed at understanding the social and economic background of the participants. Key variables collected included marital status, sex, age group and income, which provided an understanding of the caregivers’ socioeconomic and personal demographics. In addition, variables such as whether the caregivers had quit their job to look after the stroke survivor, number of children, dependent children, relationship with stroke survivor, level of anxiety or depression were also collected.

#### Stroke survivor demographic variables

The stroke survivor demographic data focused on understanding the characteristics and health status of those receiving care. Collected variables included sex, educational attainment, marital status, actual age, which are basic but essential indicators of the stroke survivor’s social and personal background. Furthermore, data on comorbidities, accommodation status, dependence, physical and social outcomes, as well as the presence of anxiety or depression were collected. These variables helped in understanding the potential demands placed on caregivers and how these might contribute to the perceived burden.

### Data analysis

Descriptive statistics, including frequency distribution, median (interquartile range [IQR]) and percentages, were used to summarise the demographic characteristics of caregivers and stroke survivors. Factors associated with CBS were tested using the Wilcoxon Rank-Sum test and the Kruskal–Wallis test in the bivariate analysis. Factors with *p* < 0.20 were considered for inclusion in the multiple linear regression analysis. Multiple linear regression analysis was conducted to determine the association between sociodemographic and stroke-related clinical variables with CBS. The Variance Inflation Factor test was conducted to check for multicollinearity at a cut-off level of 5. Variables with a *p*-value < 0.05 were considered significantly associated with CBS in the multiple linear regression analysis. The final main effects model was refined through an iterative process of removing, refitting and verifying variables until only those with significant associations were retained.

**FIGURE 2:**
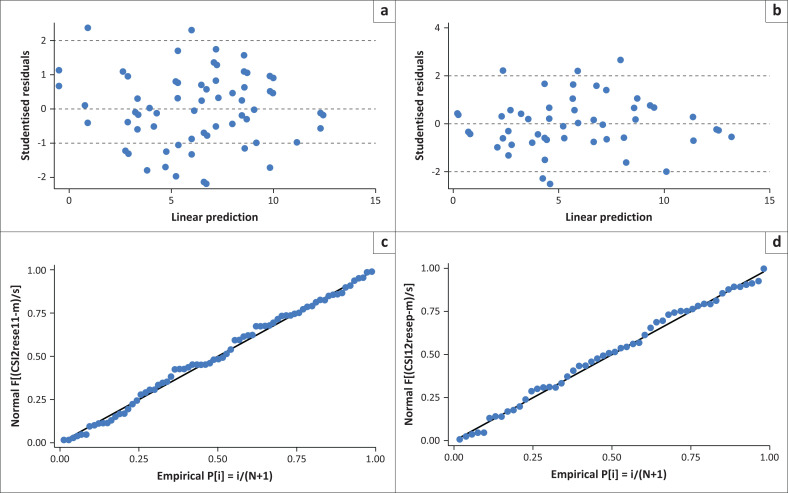
Model diagnostics at 3 months and 12 months for caregiver factors. (a & c) Residual versus fitted values plot for CBS model at 3 months. (b & d) Residual versus fitted values plot for CBS model at 12 months.

**FIGURE 3:**
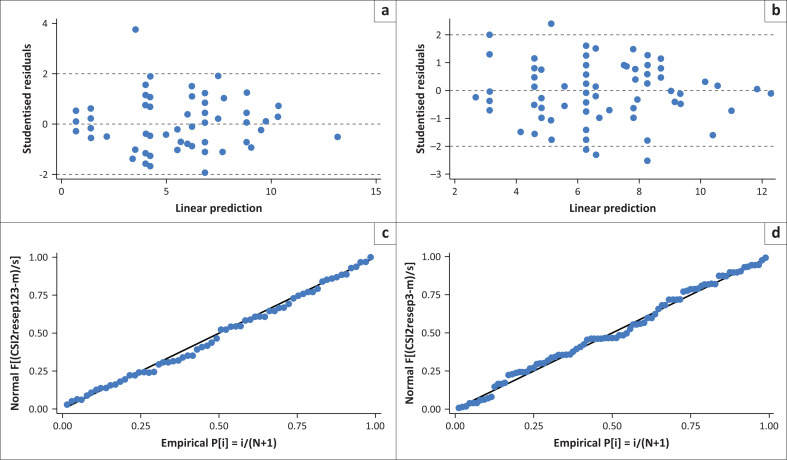
Model diagnostics at 3 months and 12 months for patient factors. (a & c) Residual vs fitted values plot for CBS model at 12 months. (b & d) Residual vs fitted values plot for CBS model at 3 months.

Model diagnostics were checked using the scatter plot of residuals versus fitted values and quantile-quantile (Q-Q) plots of residuals (Figure 2 & Figure 3). Model goodness of fit was assessed using the Akaike’s information criterion (AIC) and the linear determination index *R*^2^. Adjusted linear regression coefficients (β) with 95% confidence intervals (CI) were used to express the associations.

### Ethical considerations

Ethical approval was obtained from the Medical Research Council of Zimbabwe (MRCZ – 34/78). Informed written consent was obtained from all participants before data collection. Caregiver consent was obtained for stroke survivors who were not lucid.

## Results

### Stroke survivor participant characteristics

Table 1 presents stroke survivor participant characteristics and their association with CBS at 3- and 12-month post-stroke. The proportion of caregivers experiencing high CBS was greater among those attending to female stroke survivors (62.1%) at 3 months with a median CBS score of 8 (IQR: 4, 11), compared to those tending to male stroke survivors (37.9%), with a median CBS score of 6 (IQR: 3, 9). Similarly, caregivers experiencing high CBS were among those tending to stroke survivors not in union (56.9%) at 12 months, with a median CBS score of 7 (IQR: 4, 10), compared to those tending to married stroke survivors (43.1%) with a median score of 3 (IQR: 1, 7). Caregivers tending to stroke survivors with poor physical health outcomes (55.2%) reported a higher median CBS score of 9 (IQR: 6, 11) at 3 months, which further increased to 10 (IQR: 4, 11) at 12 months, compared to those looking after stroke survivors with good outcomes, with median scores of 5 (IQR: 2, 8) at 3 months and 4 (IQR: 1, 7) at 12 months. Similarly, poor social outcomes were associated with elevated CBS, with median CBS scores of 8 (IQR: 5, 10) versus 6 (IQR: 3, 9) at 3 months and 9 (IQR: 5, 11) versus 3 (IQR: 1, 7) at 12 months. Caregivers attending to stroke survivors with very severe motor impairments had the highest CBS median score of 11 (IQR: 9, 12) at 3 months. In addition, caregivers providing care to stroke survivors with poor community reintegration reported high median CBS scores of 9 (IQR: 5, 11) at 3 months and 9 (IQR: 6, 11) at 12 months compared to those with good community reintegration.

**TABLE 1:** Stroke survivor participant characteristics.

Characteristic	Categories	CBS at 3 months (*n* = 87)					CBS at 12 months (*n* = 65)				
		Frequency (*n*)	%	Median	IQR	*p*	Frequency (*n*)	%	Median	IQR	*p*
Sex	Male	33	37.9	6	3, 9	0.064	24	36.9	6	3, 9	0.927
	Female	54	62.1	8	4, 11	-	41	63.1	6	2, 9	-
Education	None or primary	26	30.2	8	2, 10	0.098	14	21.9	9	2, 10	0.216
	Secondary	52	60.5	8	5, 11	-	45	70.3	5	3, 8	-
	Tertiary	8	9.3	4	3, 6	-	5	7.8	2	1, 4	-
Marital status	Not in union†	47	54.0	7	4, 10	0.657	37	56.9	7	4, 10	0.006
	Married	40	46.0	7	3, 10	-	28	43.1	3	1, 7	-
Age group (years)	Below 45	31	35.6	7	3, 11	0.694	25	38.5	5	3, 8	0.774
	45–54	19	21.9	6	3, 9	-	16	24.6	6	2, 10	-
	55 and above	37	42.5	7	3, 10	-	24	36.9	7	2, 10	-
Comorbidities	No	22	25.3	6	3, 9	0.488	13	20.0	4	1, 8	0.314
	Yes	65	74.7	7	4, 10	-	52	80.0	6	3, 10	-
	Single	44	67.7	7	4, 11	-	36	69.2	6	3, 10	-
Accommodation	Own house	45	51.7	6	3, 9	0.157	36	55.4	5	2, 10	0.707
	Tenant	16	18.4	7	4, 10	-	11	16.9	7	5, 9	-
	Staying with relative	26	29.9	9	4, 11	-	18	27.7	5	2, 9	-
Patients’ bill payment method	Cash	67	77.0	7	3, 9	0.282	48	73.8	5	2, 9	0.105
	Health insurance	10	11.0	8	5, 11	-	8	12.3	7	5, 11	-
	Free service (old age/pensioners/retirees)	10	11.0	10	5, 11	-	9	13.9	8	7, 12	-
Dependence	Independent	37	42.5	6	2, 9	0.006	20	31.3	4	1, 7	< 0.001
	Dependent	50	57.5	9	6, 11	-	44	68.7	10	6, 11	-
Physical outcome	Good	39	44.8	5	2, 8	< 0.001	41	64.1	4	1, 7	< 0.001
	Poor	48	55.2	9	6, 11	-	23	35.9	10	4, 11	-
Social outcome	Good	32	36.8	6	3, 9	0.068	37	57.8	3	1, 7	< 0.001
	Poor	55	63.2	8	5, 10	-	27	42.2	9	5, 11	-
Community reintegration	Good	42	48.3	6	2, 8	0.012	39	60.9	3	1, 7	< 0.001
	Poor	45	51.7	9	5, 11	-	25	39.1	9	6, 11	-
Motor severity	Mild	54	62.1	6	2, 9	0.001	48	75.0	5	2, 7	< 0.001
	Moderate	16	18.4	7	5, 9	-	8	12.4	7	4, 11	-
	Severe	6	6.9	10	6, 10	-	4	6.3	10	8, 10	-
	Very severe	11	12.6	11	9, 12	-	4	6.3	12	12, 12	-
Functional problems	Mild	57	65.5	6	2, 9	< 0.001	49	76.6	4	1, 7	< 0.001
	Moderate	16	18.4	6	4, 9	-	9	14.1	8	5, 10	-
	Severe	6	6.9	9	9, 10	-	2	3.1	10	9, 10	-
	Very severe	8	9.2	12	11, 12	-	4	6.2	12	11, 12	-

Note: *n* = 87 at 3 month and *n* = 65 at 12 months.

IQR, interquartile range; CBS, caregiver burden strain.

† , Not in union includes, single, widowed, divorced and separated.

**FIGURE 4:**
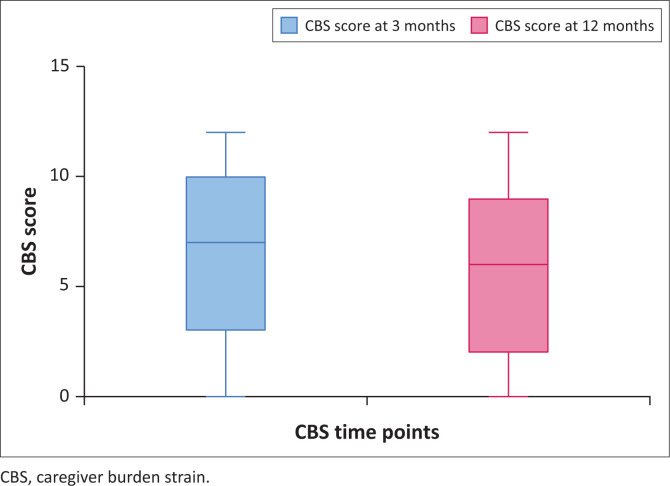
Overall care burden strain score at 3 and 12 months.

Overall, CBS was higher during the first 3 months compared to the 12-month time point (Figure 4).

### Caregiver characteristics

Results show that CBS was higher among caregivers who chose to resign from their jobs to provide care at both 3-months (median 11 [IQR: 9, 12] vs 6 [IQR: 3, 9]) and 12-months (median 7 [IQR: 5, 10] vs 5 [IQR: 2, 9]) time points compared to those who chose not to resign. Similarly, CBS was higher among those who reported feeling completely overwhelmed at both time points compared to those who were not completely overwhelmed, with CBS median scores of 9 (IQR: 6, 11) versus 4 (IQR: 2, 7) and 9 (IQR 6, 11) versus 3 (IQR: 1, 4) indicating a consistently higher CBS over time at 3 months and 12 months, respectively. In addition, CBS was high among caregivers who adjusted their work commitments compared to those who did not, with CBS median scores of 9 (IQR 6, 11) versus 5 (IQR: 2, 8) and 9 (IQR 7, 12) versus 4 (IQR: 1, 7) at 3 months and 12 months, respectively. Furthermore, caregivers with anxiety or depression problems had high CBS compared to those with no anxiety or depression problems at both time points, with median CBS scores of 9 (IQR 6, 10) at 3 months and 10 (IQR 7, 12) at 12 months (Table 2).

**TABLE 2:** Caregiver participant characteristics.

Characteristic	Categories	CBS at 3 months (*n* = 87)					CBS at 12 months (*n* = 65)				
		Frequency (*n*)	%	Median	IQR	*p*	Frequency (*n*)	%	Median	IQR	*p*
Sex	Male	27	31.0	7	3, 11	0.958	23	35.4	5	1, 8	0.397
	Female	60	69.0	7	3, 10	-	42	64.6	6	3, 10	-
Hospital	Chitungwiza hospital	25	28.7	3	2, 8	0.001	12	18.5	2	1, 5	0.005
	Sally Mugabe hospital	35	40.3	9	6, 11	-	34	52.3	7	4, 10	-
	Parirenyatwa hospital	27	31.0	6	4, 9	-	19	29.2	4	1, 9	-
Marital status	Married	65	74.7	7	3, 10	0.958	49	75.4	6	3, 9	0.307
	Not in union†	22	25.3	7	4, 9	-	16	24.6	5	1, 7	-
Age group (years)	Below 45	51	63.0	7	3, 10	0.960	41	66.1	6	3, 9	0.941
	45–54	14	17.3	8	2, 11	-	12	19.4	5	3, 9	-
	55 and above	16	19.7	8	4, 10	-	9	14.5	7	0, 10	-
Occupation	Employed full time	34	39.1	7	2, 10	0.569	26	40.0	6	3, 9	0.119
	Employed part time	27	31.0	6	3, 10	-	20	30.8	7	3, 10	-
	Informally employed	22	25.3	8	6, 11	-	16	24.6	4	2, 7	-
	Pensioner	4	4.6	7	2, 10	-	3	4.6	9	9, 12	-
Income	Comfortable	5	5.8	2	0, 5	0.080	4	6.2	1	0, 7	0.070
	Just have enough to make it	41	47.1	7	3, 9	-	27	41.5	4	2, 7	-
	Do not have enough to make it	41	47.1	8	4, 11	-	34	52.3	7	4, 10	-
Quit job to look after patient	Yes	12	14.6	11	9, 12	< 0.001	11	18.0	7	5, 10	0.307
	No	70	85.4	6	3, 9	-	50	82.0	5	2, 9	-
Number of children	≤ 3	52	67.5	8	4, 10	0.243	38	66.7	6	3, 10	0.910
	> 3	25	32.5	6	3, 9	-	19	33.3	7	1, 9	-
Dependent children	None	20	27.4	6	2, 9	0.240	11	20.0	9	1, 10	0.789
	< 3	32	43.8	8	5, 11	-	25	45.5	5	3, 7	-
	≥ 3	21	28.8	6	3, 11	-	19	34.5	4	1, 8	-
Relationship to patient	Spouse	34	39.1	8	4, 11	0.386	28	43.1	7	5, 10	0.051
	Child	26	29.9	7	4, 10	-	18	27.7	4	1, 8	-
	Relative	27	31.0	6	2, 10	-	19	29.2	4	1, 7	-
Accommodation	Owned	49	56.4	7	3, 10	0.637	35	53.8	6	2, 10	0.854
	Rented	19	21.8	8	5, 10	-	12	18.5	6	2, 8	-
	Staying with relative	19	21.8	6	3, 11	-	18	27.7	6	3, 9	-
Anxiety/depression	No problem	48	55.2	6	2, 8	0.011	45	70.3	4	1, 7	< 0.001
	Problem	39	44.8	9	6, 10	-	19	29.7	10	7, 12	-
Made work adjustment	No	48	55.2	5	2, 8	< 0.001	46	70.8	4	1, 7	< 0.001
	Yes	39	44.8	9	6, 11	-	19	29.2	9	7, 12	-
Completely overwhelmed	No	36	41.4	4	2, 7	< 0.001	30	46.2	3	1, 4	< 0.001
	Yes	51	58.6	9	6, 11	-	35	53.8	9	6, 11	-

Note: *n* = 87 at 3 months and *n* = 65 at 12 months.

CBS, caregiver burden strain; IQR, interquartile range.

† , Not in union includes, single, widowed, divorced and separated.

### Caregiver factors associated with caregiver burden

In multiple linear regression analysis, after adjusting for other variables, caregivers at Sally Mugabe Hospital experienced a significantly higher CBS at 3 months (β = 1.85, 95% CI: 0.39–3.31, *p* = 0.014) and at 12 months (β = 2.86, 95% CI: 0.07–5.66, *p* = 0.045), compared to those at Chitungwiza Hospital. Similarly, caregivers from Parirenyatwa Hospital showed a higher CBS at 3 months (β = 1.97, 95% CI: 0.52–3.41, *p* = 0.008) compared to the reference hospital. Caregivers reporting anxiety or depression had a significantly higher CBS at both 3 months (β = 2.46, 95% CI: 1.30–3.61, *p* < 0.001) and 12 months (β = 2.73, 95% CI: 0.54–4.91, *p* = 0.016). Making work adjustments because of caregiving was associated with a significantly higher CBS at 3 months (β = 3.84, 95% CI: 2.71–4.97, *p* < 0.001) compared to no work adjustments. Caregivers reporting feeling completely overwhelmed had a significantly higher CBS at 3 months (β = 3.36, 95% CI: 2.10–4.62, *p* < 0.001) compared to those not completely overwhelmed (Table 3).

**TABLE 3:** Caregiver factors associated with caregiver burden strain.

Variable	CBS at 3 months			CBS at 12 months		
	β	95% CI	*p*	β	95% CI	*p*
**Hospital**						
Chitungwiza Hospital	Reference	-	-	-	-	-
Sally Mugabe Hospital	1.85	0.39, 3.31	0.014	2.86	0.07, 5.66	0.045
Parirenyatwa Hospital	1.97	0.52, 3.41	0.008	2.45	−0.78, 5.68	0.133
**Dependent children**						
None	Reference	-	-	-	-	-
< 3	1.28	−0.08, 2.64	0.065	NS	-	-
≥ 3	1.41	−0.08, 2.90	0.063	NS	-	-
**Anxiety/depression**						
No problem	Reference	-	-	-	-	-
Problem	2.46	1.30, 3.61	< 0.001	2.73	0.54, 4.91	0.016
**Made work adjustments**						
No	Reference	-	-	-	-	-
Yes	3.84	2.71, 4.97	< 0.001	1.19	−0.92, 3.29	0.262
**Completely overwhelmed**						
No	Reference	-	-	-	-	-
Yes	3.36	2.10, 4.62	< 0.001	1.86	−0.74, 4.47	0.157

Note: *n* = 87 at 3 months and *n* = 65 at 12 months. Multiple linear regression.

β, multiple linear regression coefficient; NS, not selected for multiple linear regression; CI, confidence interval; CBS, caregiver burden strain.

### Stroke survivor characteristics associated with caregiver burden

**FIGURE 5:**
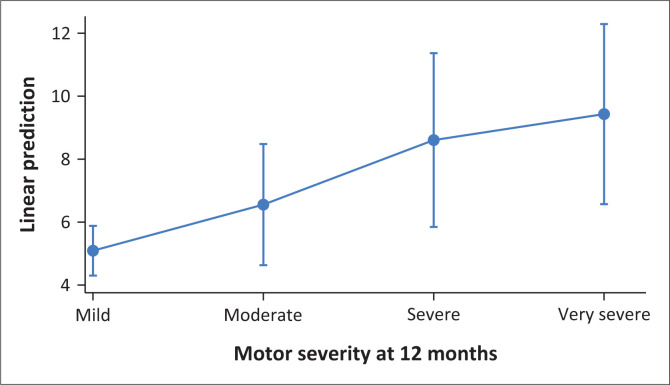
Predictive margins of motor severity at 12 months with 95% confidence interval.

**FIGURE 6:**
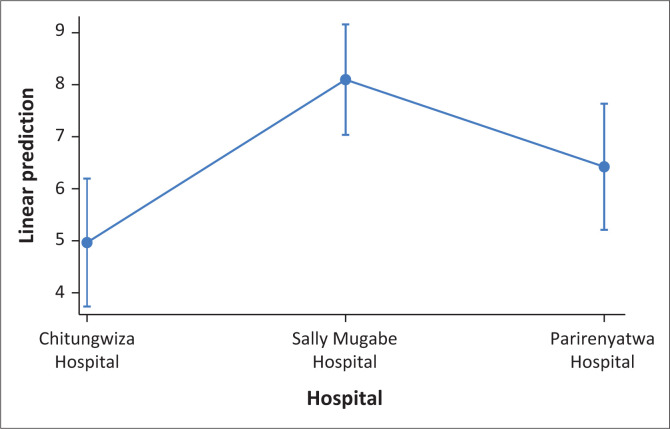
Predictive margins of hospitals at 12 months with 95% confidence interval.

At 3 months, caregivers providing care to stroke survivors at Sally Mugabe Hospital reported a significantly higher CBS (β = 3.13, 95% CI: 1.50–4.76, *p* < 0.001) compared to those at Chitungwiza Hospital. Similarly, at 12 months, caregivers at Sally Mugabe Central Hospital also showed a significantly elevated CBS (β = 3.31, 95% CI: 1.39–5.24, *p* < 0.001). Similarly, caregivers tending to stroke survivors with poor physical outcomes reported a significantly increased CBS at 3 months (β = 2.00, 95% CI: 0.31–3.70, *p* = 0.021), compared to those attending to stroke survivors with good outcomes. Furthermore, caregivers tending to stroke survivors using health insurance had a higher CBS at 3 months (β = 2.44, 95% CI: 0.33–4.55, *p* = 0.024), compared to those using cash payments. At 12 months, attending to married stroke survivors was associated with a significant reduction in CBS (β = –2.83, 95% CI: –4.23 to –1.43, *p* < 0.001). Stroke survivors with motor severity categories of severe (β = 3.51, 95% CI: 0.61–6.41, *p* = 0.018) and very severe (β = 4.34, 95% CI: 1.33–7.35, *p* = 0.006) were significantly associated with an increased CBS at 12 months. In addition, caregivers providing care to stroke survivors with poor social outcomes reported a significantly higher CBS at 12 months (β = 1.99, 95% CI: 0.47–3.51, *p* = 0.011) (see Table 4, Table 5, Figure 5, Figure 6).

**TABLE 4:** Stroke survivor participant characteristics associated with caregiver burden.

Variable	CBS at 3 months			CBS at 12 months		
	β	95% CI	*p*	β	95% CI	*p*
**Hospital**						
Chitungwiza Hospital	Reference	-	-	-	-	-
Sally Mugabe Hospital	3.13	1.50, 4.76	< 0.001	3.31	1.39, 5.24	< 0.001
Parirenyatwa Hospital	1.46	−0.27, 3.18	0.097	0.70	−1.35, 2.76	0.496
**Physical outcome**						
Good	Reference	-	-	-	-	-
Poor	2.00	0.31, 3.70	0.021	NS	-	-
**Functional problems**						
Mild	Reference	-	-	-	-	-
Moderate	−0.32	−2.40, 1.76	0.761	NS	-	-
Severe	2.74	−0.12, 5.61	0.060	NS	-	-
Very severe	4.02	1.41, 6.63	0.003	NS	-	-
Patients’ bill payment method						
Cash	Reference				-	
Health insurance	2.44	0.33, 4.55	0.024	NS	-	-
Free service (old age/pensioners/retirees)	−0.45	−2.63, 1.72	0.678	NS	-	-
**Patients’ marital status**						
Single	Reference	-	-	-	-	-
Married	NS	-	-	−2.83	−4.23, -1.43	< 0.001
**Motor severity**						
Mild	Reference	-	-	-	-	-
Moderate	NS	-	-	1.46	−0.64, 3.57	0.170
Severe	NS	-	-	3.51	0.61, 6.41	0.018
Very severe	NS	-	-	4.34	1.33, 7.35	0.006
**Social outcome**						
Good	Reference	-	-	-	-	-
Poor	NS	-	-	1.99	0.47, 3.51	0.011

Note: *n* = 87 at 3 months and *n* = 65 at 12 months. Multiple linear regression.

β, Multiple linear regression coefficient, NS, Not selected for multiple linear regression CI, confidence interval; CBS, caregiver burden strain.

**TABLE 5:** Caregiver burden strain model equations.

Timepoint	Model equations for caregiver factors	Model equations for patient factors
CBS at 3 Months	$$\begin{array}{l} {\text{CBS}}_{3\;months}=\beta_{0}+1.85\times (\text{Sally Mugabe})+1.97\times (\text{Parirenyatwa})+2.46\times \\ (\text{Anxiety/Depression})+3.84\times (\text{Made Work Adjustments})+3.36\times \\ \left(\text{Completely Overwhelmed}\right) \end{array}$$ [Eqn 1]	$$\begin{array}{l} {\text{CBS}}_{3\;months}\text{s}=\beta_{0}+3.13\text{v}\times \text{Sally Mugabe}+4.02\times \text{Very Severe Functional}\\ \text{Problems}+2.00\times \text{Poor Physical Outcome}+2.44\times \text{Health Insurance}\\ \text{Payment}+3.51\times \text{Severe Motor Severity}+4.34\times \text{Very Severe Motor}\\ \text{Severity} \end{array}$$ [Eqn 2]
CBS at 12 Months	$$\begin{array}{l} {\text{CBS}}_{12\;months}=\beta_{0}+2.86\times (\text{Sally Mugabe})+2.73\times \\ \left(\text{Anxiety/Depression}\right) \end{array}$$ [Eqn 3]	$$\begin{array}{l} {\text{CBS}}_{12\;months}=\beta_{0}+3.31\times \text{Sally Mugabe}-2.83\times \text{Married Status}+4.34\times \text{Very}\\ \text{Severe Motor Severity}+1.99\times \text{Poor Social Outcome} \end{array}$$ [Eqn 4]
Key	Where: β_**0**_ represents the intercept. Sally Mugabe and Parirenyatwa are dummy variables for hospital categories. Anxiety/depression, made work adjustments and completely overwhelmed are binary variables indicating the presence of the conditions:	Where: β_**0**_ represents the intercept. Sally Mugabe Hospital, very severe functional problems, poor physical outcome, health insurance payment, severe motor severity, very severe motor severity, married status and poor social outcome are dummy variables indicating the presence of conditions.

CBS, caregiver burden strain.

## Discussion

The results of our study highlight CBS following stroke, revealing significant associations between caregiver and stroke survivor factors and CBS at both 3- and 12-month post-stroke follow-up. These factors highlight the multi-faceted nature of CBS in the Zimbabwean context. These factors were critical in predicting the level of CBS among our participants. Understanding these predictive relationships is essential for developing targeted interventions to mitigate CBS.

Caregivers experiencing feelings of being overwhelmed, anxious or depressed were found to be at increased risk of CBS. This demonstrates the critical role of caregiver mental health in burden perception. Our findings are similar to those of other studies that indicated that caregivers’ psychological well-being significantly influences their ability to cope with caregiving responsibilities (Pinquart & Sörensen 2003). The stronger association observed between feeling overwhelmed and CBS at 12 months compared to 3 months suggests that caregiver burden may intensify over time, necessitating ongoing support and intervention strategies (Patel et al. 2013). This may be due to the extensive and continuous care needs of stroke survivors resulting in caregivers experiencing a sense of being overwhelmed overtime. This can stem from the emotional toll of watching a loved one struggle with impairments as well as the physical demands of caregiving (Camak 2015). The feeling of being overwhelmed among the caregivers can indicate that the demands of caregiving exceeded their coping capacity (Adelman et al. 2014).

Depression and anxiety were also found to be a predictor of CBS in our study, a sign that mental health of the caregivers is closely linked to their CBS. Depression and anxiety are often a result of social isolation, concerns about the future and the stress associated with caregiving responsibilities (Rigby, Gubitz & Phillips 2009). This can reduce the caregiver’s ability to effectively provide care, leading to a vicious cycle of worsening mental health and increasing CBS making this relationship bidirectional in nature. Interventions aimed at early identification, reducing the complexity of care or providing additional support can be effective in alleviating this feeling of being overwhelmed, thereby lowering the overall CBS (Katon & Schulberg 2016; Rigby et al. 2009).

Our findings also highlighted the fact that caregivers may have left their jobs to provide full-time care, leading to financial strain. This decision to leave a job to provide care may be a demonstration of the severity of the stroke survivor’s condition, which correlates with a higher CBS among our participants. This loss of income, combined with the costs associated with stroke care, may have intensified stress and added to the caregiver’s overall CBS (Haley et al. 2015). According to Glozman (2016), economic strain resulting from loss of employment is a powerful predictor of CBS. To help reduce CBS, financial support mechanisms or flexible work arrangements may need to be put in place (Moon & Dilworth-Anderson 2015).

While health insurance is generally seen as beneficial, in our context, it was associated with increased CBS. This counterintuitive finding could be because of limitations of insurance coverage, requiring caregivers to still manage significant out-of-pocket expenses or navigate complex healthcare systems, which can be stressful (Covinsky et al. 2011; Rigby et al. 2009). This factor underscores the importance of simplifying insurance processes and ensuring comprehensive coverage to reduce CBS (Brodaty, Green & Koschera 2003).

Our findings may have been influenced by a mixture of socioeconomic standing and patient-associated factors. The management of stroke survivors in Zimbabwe should be multifactorial with the involvement of several health professional stakeholders. A previously published study retrospectively investigated the needs of caregivers and patients with stroke (Kaseke et al. 2017) and different caregivers reported facing challenges during daily activities such as bathing, toileting and mobilisation. However, the caregiver burden was not measured. Our current study therefore quantified this problem and predicts the caregivers who may have higher CBS based on several factors as a way to manage CBS.

Caregivers providing care to stroke survivors who were not in a union showed significantly higher CBS median scores compared to caregivers attending to married stroke survivors. This may suggest that stroke survivor marital status may play a crucial role in determining the level of support required from caregivers, with those lacking a partner potentially increasing greater caregiving responsibilities for the caregivers, such as children and siblings (Epping-Jordan & WHO Global Report on Effective Interventions for Non-Communicable Diseases 2014). Furthermore, caregivers who are not spouses may find themselves with fewer resources and less emotional support, which increases their CBS (Adelman et al. 2014). This factor indicates that the availability of a supportive partner is protective against high levels of CBS, and interventions could focus on strengthening social support networks for caregivers who are not in union (Evercare Study 2006). However, some caregivers in our study were spouses to the stroke survivors and had significant CBS suggesting that caring for a spouse may affect caregiver burden.

Caregivers attending to older stroke survivors aged 55 years and above were vulnerable to increased CBS. As older age is often associated with declining overall health and increasing susceptibility to co-morbid long-term conditions, a stroke may exacerbate an already pre-existing high CBS and add to challenges faced by caregivers of older adult stroke survivors (Carod-Artal et al. 2000). Given the increasing prevalence of stroke among older adults and the associated care requirements, there is a need for targeted support services tailored to older caregivers along the trajectory of care in Zimbabwean stroke survivors (Garnett et al. 2022). Most of the older survivors had no source of income and therefore could not contribute to their upkeep thereby increasing caregiver burden.

Poor functional outcomes and poor community reintegration among the stroke survivors increased CBS in our participants especially during the first 3 months. This is because poor function increases dependency and extended care leading to poor community reintegration of the survivor, thereby escalating the caregiver’s workload (Haley et al. 2015; Jaracz, Grabowska-Fudala & Kozubski 2015; Lutz & Young 2010b; Oyewole & Odebiyi 2017). Stroke survivors who struggle to reintegrate into the community often place a higher burden on their caregivers as reported in our findings. Poor community reintegration also predicts greater CBS because it signifies ongoing dependency and the need for long-term care. Effective community-based programs that facilitate reintegration and promote independence can therefore be crucial in reducing the long-term burden on caregivers (Smith & Harkness 2016). In addition, rehabilitation programs that enhance motor outcomes and function are crucial in reducing caregiver burden strain, as CBS tends to increase proportionally with a survivor’s loss of independence (Lutz & Young 2010b).

Our results have implications for the development of targeted interventions aimed at alleviating caregiver burden and improving outcomes for both caregivers and stroke survivors. Interventions focused on enhancing caregiver coping strategies and resilience (Qureshi et al. 2022a, 2022b), providing social support networks and facilitating access to mental health services may help mitigate the impact of caregiver psychological distress on CBS (King et al. 2010). Interventions targeting patient outcomes, such as rehabilitation programmes aimed at improving physical and social functioning, may contribute to reducing CBS by enhancing stroke survivor independence thus reducing caregiver responsibilities (Bindawas & Vennu 2016; Cawood & Visagie 2016; Haley et al. 2011). Our findings highlight the importance of early screening and assessment of caregiver and stroke survivor needs to identify those at increased risk of CBS to provide timely support and resources (Janssen et al. 2014). By addressing both caregiver and stroke survivor factors, healthcare providers can better support caregivers and stroke survivors, ultimately improving outcomes and quality of life for both groups in the post-stroke period. However, it is important to note that it may not be easy to improve the outcome of stroke survivors. This is because 60% of stroke survivors acquire permanent disabilities and experience limitations in terms of mobility, vision, voice, speech, swallowing and sexual function, which contribute to long-term CBS (Kalavina et al. 2019). Although rehabilitation may not reverse the brain damage, it can substantially help people achieve the best possible long-term outcomes through facilitation of neuroplasticity of the brain (Mlambo et al. 2021).

It is also important to acknowledge that while being in a LMIC like Zimbabwe can present unique challenges such as limited healthcare resources, economic constraints and social support systems, this does not necessarily mean that stroke survivors and their caregivers face more challenges compared to those in high-income countries (Oyewole & Odebiyi 2017). Caregiver burden strain is influenced by various factors, including cultural values, family structures and community support, which can mitigate or exacerbate CBS depending on the context (Epping-Jordan & WHO Global Report on Effective Interventions for Non-Communicable Diseases 2014). The strong familial and community ties found in many LMICs like Zimbabwe may provide a level of support that can overshadow the impact of CBS. Furthermore, traditional caregiving roles often seen in these settings can sometimes help caregivers manage the challenges they face more effectively than might be expected in high-income countries, where social isolation can be more pronounced (Epping-Jordan & WHO Global Report on Effective Interventions for Non-Communicable Diseases 2014; Haley et al. 2015).

### Strengths and limitations

The longitudinal study design and the use of multiple time points allowed for an understanding of CBS over time. Response bias may have been introduced from self-reporting, as participants may under-report or over-report their experiences. Furthermore, attrition over the course of the longitudinal study may have affected the validity of the findings, as the loss of participants could have introduced selection bias. Our study might have been underpowered because of attrition as indicated by wide 95% confidence intervals, which show a low level of precision.

### Clinical implications

Comprehensive and multi-faceted interventions that address the mental health needs of caregivers, enhance social support systems and improve stroke outcomes to effectively reduce CBS and improve the well-being of both caregivers and stroke survivors in resource-constrained settings like Zimbabwe are needed.

## Conclusion

Our study highlights the significant impact of both caregiver and stroke survivor factors on CBS experienced by individuals caring for stroke survivors in Zimbabwe, particularly at Sally Mugabe Hospital, where the CBS was significantly higher at both 3 months and 12 months post-stroke. The burden was also notably elevated among caregivers tending to stroke survivors at Parirenyatwa Hospital at 3 months. Caregivers experiencing anxiety or depression, those who made work adjustments and those who felt completely overwhelmed reported significantly increased CBS, particularly in the early stages of caregiving. Furthermore, caregivers providing care to stroke survivors with poor physical outcomes and those with severe or very severe motor impairments faced a greater burden over time. Interestingly, caring for married stroke survivors was associated with a reduced CBS at 12 months, while poor social outcomes were linked to a higher burden. The predictive nature of these factors highlights the importance of early intervention and targeted support for both caregivers and stroke survivors. By providing mental health support to caregivers, financial assistance and improving rehabilitation outcomes for stroke survivors, healthcare providers can better manage and reduce CBS. Additionally, understanding these predictive relationships allows for the development of personalised care plans that consider both the caregiver’s and the stroke survivor’s needs, ultimately leading to better outcomes for both parties.

These findings suggest that caregivers and stroke survivors might benefit from continued engagement with supportive services.
